# National recommendations of the Croatian society of medical biochemistry and laboratory medicine: Thyroid function tests from the laboratory point of view

**DOI:** 10.11613/BM.2025.030505

**Published:** 2025-10-15

**Authors:** Adriana Bokulić, Ivana Zec, Domagoj Marijančević, Marija Siter Kuprešanin, Sanja Goreta, Anamarija Đuras, Koraljka Đurić, Sanda Jelisavac Ćosić, Iva Lukić, Tihana Serdar Hiršl, Lada Stanišić, Daniela Šupe-Domić, Alenka Pezo, Marija Kocijančić

**Affiliations:** 1 Department of Clinical Chemistry, Sestre Milosrdnice University Hospital Center, Zagreb, Croatia; 2 School of Medicine, Catholic University of Croatia, Zagreb, Croatia; 3 Department of Medical Laboratory Diagnostics, University Hospital Sveti Duh, Zagreb, Croatia; 4 Department of Medical Biochemistry Laboratory, General Hospital Varaždin, Varaždin, Croatia; 5 Department of Clinical Laboratory Diagnostics, Srebrnjak Children’s Hospital, Zagreb, Croatia; 6 Department of Nuclear Medicine and Radiation Protection, University Hospital Centre Zagreb, Croatia; 7 Clinical Institute of Laboratory Diagnostics, University Hospital Centre Osijek, Osijek, Croatia; 8 Faculty of Medicine Osijek, JJ Strossmayer University of Osijek, Osijek, Croatia; 9 Synlab Croatia, Polyclinic for Medical Laboratory Diagnostics, Zagreb, Croatia; 10 Department of Medical Laboratory Diagnostics, University Hospital of Split, Split, Croatia; 11 Department of Health Studies, University of Split, Split, Croatia; 12 Medical Biochemistry Laboratory Alenka Pezo, Zagreb, Croatia; 13 Institute for Clinical Chemistry and Pathobiochemistry, Department for Diagnostic Laboratory Medicine, University Hospital Tübingen, Tübingen, Germany

**Keywords:** thyroid function tests, standardization, recommendations

## Abstract

Thyroid function tests (TFTs) - thyroid stimulating hormone (TSH), total triiodothyronine (T3), total thyroxine (T4), free triiodothyronine (FT3), free thyroxine (FT4), thyroid peroxidase antibodies (anti-TPO), thyroglobulin antibodies (anti-Tg), TSH receptors antibodies (anti-TSHR), and thyroglobulin (Tg) - are used to diagnose thyroid disorders and are crucial biomarkers for monitoring and managing thyroid cancer. The 2022 national survey results revealed that thyroid function testing is not standardized among Croatian medical-biochemistry laboratories. Laboratories follow individual protocols at each testing stage, from patient preparation to result reporting. To address this, the Working group for laboratory endocrinology of the Croatian society of medical biochemistry and laboratory medicine has developed recommendations based on the latest national and international guidelines, research and the authors’ expert opinion. The document aims to standardize all phases of thyroid function testing, with 7 preanalytical, 12 analytical, and 8 postanalytical recommendations, each supported by expert explanations. While primarily directed at Croatian laboratory professionals, this document is also relevant to other healthcare professionals managing thyroid-related health issues.

## Graphical abstract

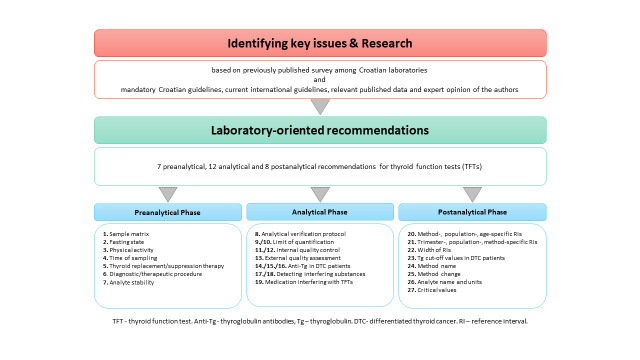

## Highlights

• The previously published survey revealed significant variability in practices regarding thyroid function tests among more than 250 Croatian medical-biochemistry laboratories

• The recommendations address key aspects of routine laboratory work, considering local Croatian possibilities within the national healthcare system

• Seven preanalytical, 12 analytical and 8 postanalytical recommendations are based on mandatory national guidelines, published research, international guidelines, and consensus among the authors

• The aim is to provide a laboratory-oriented framework that harmonizes practices and policies to benefit not only laboratories but also patients and their physicians

## Introduction

Thyroid function tests (TFTs) are essential for evaluating thyroid gland disorders. These tests may include the measurement of thyroid stimulating hormone (TSH), total triiodothyronine (T3), total thyroxine (T4), free triiodothyronine (FT3), free thyroxine (FT4), thyroid peroxidase antibodies (anti-TPO), thyroglobulin antibodies (anti-Tg), TSH receptors antibodies (anti-TSHR), and thyroglobulin (Tg).

The most common thyroid disorders in adults include clinical and subclinical hypo- or hyperthyroidism, often of autoimmune origin, as well as thyroid carcinomas (*1*-*3*). Given the role of thyroid hormones in the growth and development of the entire organism, poor thyroid gland function can result in significant issues, especially in pregnancy, childhood, and adolescence (*4*, *5*). The concentration of TFTs can also be influenced by certain medications and may vary between fasting and postprandial states or exhibit diurnal variation (*6*-*10*). Yet, the greatest challenges in determining TFTs are related to measurement methods based on immunochemistry. Despite attempts to harmonize and standardize the results of TFTs, methods still vary depending on the manufacturer, leading to incomparable values (*11*-*15*). Additionally, immunochemistry assays are affected by interference phenomena, which may cause further complications (*16*).

Current clinically oriented guidelines do not provide comprehensive protocols and processes for analyzing TFTs in the medical-biochemistry laboratory (MBL), potentially resulting in significant variability in laboratory practices (*17*-*26*). This was demonstrated in a 2022 published Croatian survey by the Working group for laboratory endocrinology of Croatian society of medical biochemistry and laboratory Medicine (CSMBLM), which revealed considerable heterogeneity in preanalytical, analytical, and postanalytical procedures (*27*). Survey results were crucial in identifying key points and served as a foundation for developing laboratory-oriented TFT recommendations based on published research and guidelines, mandatory national guidelines from the Croatian Chamber of medical biochemists (CCMB), and the authors’ expert opinion. They are primarily intended for Croatian laboratory professionals who analyze TFTs as well as for all other medical professionals who deal with thyroid disorders. All recommendations are summarized in Appendix 1.

## Preanalytical recommendations

1. The manufacturer’s instructions for the sample matrix should be followed.

Most immunoassays used for TFTs are designed for a specific sample matrix; hence, the manufacturer’s instructions for the sample matrix must be strictly followed (*28*). Plasma tubes with heparin and ethylenediaminetetraacetic acid (EDTA) anticoagulant can be used interchangeably with serum tubes for thyroid function analytes, except for Tg and anti-TSHR (*29*). Although the World Health Organization (WHO) recommended serum samples, the difference in TSH concentrations between serum and plasma tubes had no clinical significance and was in accordance with desirable specifications for bias (*30*). Thyroid function tests determined with or without gel-containing separator tubes are comparable (*31*).

2. Blood samples for thyroid function tests should be taken in fasting state.

Recent studies indicate that TSH concentrations can be affected by food intake, with postprandial TSH being lower than the fasting value (*32*). Follow-up studies have shown that food consumption may lead to a 10-35% reduction in TSH measurements (*33*). The induced elevation of circulating somatostatin in the postprandial period and the consequent suppression of TSH could explain these results (*6*, *32*, *34*, *35*). The time factor related to circadian rhythm and the influence of food intake could dependently lead to a reduction in TSH concentrations (*33*). Kamat *et al.* proposed that the decline in TSH after eating might depend on the meal’s composition. They found that a normocaloric meal led to a postprandial TSH decrease, whereas a hypocaloric meal did not (*7*). Furthermore, when calorie intake was similar, the specific components of the calories did not significantly affect the TSH variation rate (*33*). Several studies have noted changes in FT3 and FT4 concentrations after meals, though findings on the direction of these changes are inconsistent (*7*, *32*, *36*). Consequently, patients should be fasting to prevent unclear TFT results and potential misdiagnosis of hypothyroidism.

3. Patients should refrain from strenuous physical activity 24 hours before blood sam-pling for thyroid function tests.

Physical activity can affect various metabolic and endocrine functions by increasing energy expenditure during activity and elevating the resting metabolic rate. Importantly, exercise itself can lead to hemodynamic changes, which may alter circulating hormone concentrations (*37*). Thyroid hormones are crucial for meeting the heightened metabolic demands that arise with exercise, and these hormonal fluctuations can be detected immediately after physical activity and in the following hours. It is essential to differentiate genuine hormonal concentration changes from those caused by acute, temporary hemoconcentration due to exercise, which could affect circulating TSH concentrations. Physical exercise has been shown to enhance the peripheral conversion of T4 and increase its uptake in the liver (*37*). Moreover, both cortisol and catecholamine actions initiated by exercise will also stimulate peripheral T4 deiodination (*37*). Previous studies have shown varying effects on thyroid hormone concentrations during physical exercise, with some reporting increases and others decreases. The influence of exercise on thyroid function remains debated. It appears to depend on factors such as the intensity and duration of the activity, age, dietary control, and the timing of blood sample collection post-exercise. Increased serum thyroid hormones immediately after the exercise might reflect catecholamine stimulation of hormonal secretion. Conversely, the increased heat production during exercise might activate mechanisms that lower serum thyroid hormone concentrations to prevent excessive body temperature rise. These controversial findings are thought to arise partly from misinterpretations related to changes in plasma volume after exercise, which can result in either hemoconcentration or hemodilution effects (*38*-*41*). In summary, the thyroid’s response to exercise is quite intricate, making it challenging to reach a definitive conclusion about its effects. To ensure accurate assessments, it is advisable to avoid measuring circulating hormones during periods of acute changes. Patients should refrain from strenuous physical activity 24 hours before blood sampling to minimize potential influences on hormone concentrations. This approach helps in obtaining clearer and more reliable results.

4. Blood samples should be collected between 7 and 11 AM due to the circadian rhythm of TSH and FT3.

Circulating TSH shows a typical circadian rhythm, peaking between 11 PM and 5 AM and reaching its lowest point between 5 and 8 PM (*8*, *42*). Thyroid stimulating hormone is secreted in pulses every 2 to 3 hours, alternating with periods of steady, non-pulsatile secretion. The low amplitude of the pulses, combined with TSH’s long half-life, leads to only slight variations in circulation. Over 24 hours, nocturnal TSH concentrations are roughly double the steady daytime concentrations. Research by Roelfsema *et al.* found no differences in TSH secretion patterns between men and women (*43*). Notably, infants under one month and patients with Sheehan’s syndrome do not exhibit this circadian variation (*44*). Factors such as sleep deprivation and short-term untreated hypothyroidism can disrupt the nocturnal surge of TSH. However, these biological variations are not thought to affect the diagnostic utility of TSH measurements taken between 8 AM and 6 PM (*8*, *28*, *42*, *44*, *45*). Although the mean difference in TSH concentrations due to sample timing is about 0.5 - 0.6 mIU/L, even this minor variation can be clinically significant, particularly in diagnosing subclinical hypothyroidism (SCH), pre-pregnancy counselling and addressing subfertility. Studies found a positive correlation between TSH fluctuations throughout the day and its morning concentrations, but no such correlation exists with afternoon values (*42*). This suggests that afternoon TSH measurements may not be as useful for the primary diagnosis of SCH. Additionally, studies on FT4 fluctuations show no significant circadian rhythm in hypothyroid patients (*42*). In contrast, there is a statistically significant difference between morning and afternoon FT3 concentrations, with a positive correlation between FT3 and TSH variations throughout the day (*42*). The concentration of T3 and FT3 peaks and reaches their lowest point within 0.5 to 2.5 hours after TSH. This implies that the circadian rhythm of TSH has likely a greater impact on FT3 variability than FT4 variability. A possible explanation for this could be the shorter half-life of T3 and its lower affinity for plasma proteins (*42*). The 24-hour TSH suppression patterns in thyroid cancer patients warrant further studies (*44*). Standardization is crucial for accurate determination of TFTs, and the timing of sample collection must be controlled. Blood samples should be collected between 7 and 11 AM.

5. Any thyroid replacement or suppression therapy should be administered after blood collection.

Numerous studies have concluded that patients taking exogenous levothyroxine (L-T4) have significantly higher serum T4 and FT4 concentrations compared to euthyroid control subjects without complete suppression of TSH (*46*). Interestingly, these elevated concentrations do not correlate with serum T3 and FT3 increases, resulting in a higher T4/T3 ratio. Approximately 62-82% of orally administered L-T4 is absorbed, typically within 2 hours for euthyroid individuals and around 3 hours for those with primary hypothyroidism (*47*). Free thyroxine rises in a linear fashion during the first 60-90 minutes, peaking between 2- and 4 hours post-ingestion (15 to 25%), before gradually declining back to baseline within 24 hours (*46*). Given that L-T4 has a half-life of approximately one week, the transient elevations in T4 and FT4 observed over 9 hours are merely artefacts of hormone equilibration in the serum and do not represent steady-state values. The negligible effect on T3 concentrations during this interval indicates that these early values are not relevant to metabolic status. This highlights the critical importance of timing in blood collection for outpatient management. To accurately assess thyroid hormone concentrations, it is crucial to time blood samples appropriately, ideally about 10 hours after the last administered dose. Patients are often advised to take their daily dose after collecting blood samples, which is particularly important for determining the minimum L-T4 dosage needed for TSH suppression (*46*, *47*). The European Thyroid association and the European Society for pediatric endocrinology recommend that blood samples for laboratory evaluation should be taken either before L-T4 intake or at least 4 hours after L-T4 administration (*17*, *18*, *48*). Therefore, it is best for patients to have their blood drawn before their next L-T4 dose and to take their medication afterwards. If a patient has already taken their hormone therapy before blood collection, the laboratory needs to record the timing of the last dose. Additionally, research by Ain *et al.* highlights that FT4 concentrations in L-T4 users can vary based on the time of day, indicating that the interval between L-T4 intake and blood sampling should be considered when interpreting FT4 values (*46*). This careful timing ensures more accurate assessments and effective management of thyroid hormone therapy (*18*).

Propylthiouracil (PTU) is commonly used to treat hyperthyroidism and has a significant impact on T4 deiodination. Studies indicate that both euthyroid and hyperthyroid patients experience a notable reduction (around 50%) in serum T3 concentrations within just a few hours after taking doses ranging from 50 to 300 mg of PTU (*49*). Given its rapid effect on hormone concentrations and its common use in combined therapy, it is advisable for patients to undergo suppression therapy after blood samples are collected. This timing helps ensure that hormone measurements accurately reflect the patient’s true metabolic state.

6. Patients should avoid any therapeutic and diagnostic procedures affecting thyroid func-tion tests 1 to 3 months before blood collection.

Iodinated contrast media (ICM) are widely used in radiological imaging to enhance the visibility of blood vessels, tissues, and organs by increasing the contrast between different structures. Exposure to ICM can lead to either hyperthyroidism or hypothyroidism due to the high iodine concentrations in the contrast solution, which disrupts the regulation of thyroid hormones (*19*). The immediate effect of pharmacologic doses of iodine is to inhibit the organification of iodide, reducing hormone biosynthesis and decreasing the proteolysis of Tg, which in turn lowers thyroid hormone secretion. This results in a slight decrease in serum T4 and T3, prompting a temporary increase in TSH concentrations. After the acute phase, normal thyroid hormone synthesis typically resumes, even with ongoing excess iodide exposure. However, weeks following ICM exposure, various observational studies have shown fluctuations in serum TSH, FT4, and FT3 concentrations, likely influenced by iodine intake and any pre-existing thyroid conditions. Although these changes often remain within reference intervals, they can complicate the interpretation of TFTs. The European Thyroid Association recommends that blood samples for laboratory evaluation be taken before any therapeutic or diagnostic procedures that might affect thyroid function. The laboratory needs to document this information if a procedure has already been performed before blood sampling. Additionally, when interpreting TFT results, it is crucial to consider any ICM exposure within the last 1 to 3 months (*19*, *50*).

7. The MBL should define analyte stability time for thyroid function tests.

Delayed analyses or subsequent requests are often necessary in the practice of MBLs, and it is essential to understand the stability of analytes. Research by Takac *et al.*, conducted according to the European Federation of Clinical Chemistry and Laboratory Medicine Working group for the preanalytical phase’s recommendations on stability testing, established that TSH, T3, T4, FT3, FT4, anti-Tg, and anti-TPO remain stable after centrifugation in serum gel tubes for 8 hours at room temperature or for 72 hours at 2-8 °C (*51*). Oddoze *et al.* also found that TSH, FT4, and FT3 are stable in serum gel tubes at room temperature for 24 hours before centrifugation (*52*). The Working group on preanalytical quality from the German Society for clinical chemistry and laboratory medicine reports that TSH remains stable at room temperature for three days, FT4 for five days, and FT3 for two days. However, the type of test tubes and the maximum permissible difference are not specified. The group also found that T4 is stable in serum at 2-8 °C for up to seven days and T3 for up to eight days (*53*). Manan *et al.* demonstrated that T4 and T3 are stable in serum at

2-8 °C for 72 hours after centrifugation (*54*). Additionally, Tg remains stable for at least 24 hours in unseparated serum at 2-8 °C or in separated serum at room temperature, but it is not stable in serum that has been frozen, even for a short time (*55*).

Serum and plasma samples for TFTs can be used fresh or fresh-frozen and stored at - 20 °C, - 40 °C, or - 80 °C, but repeated thaw-freeze cycles should be avoided. The stability of the glycoprotein hormone TSH is limited, and similar precautions apply to FT4 and FT3. While these molecules are stable, the thyroid hormone binding proteins can be affected by freeze-thaw cycles, which in turn impacts the concentrations of free hormone (*28*).

The MBL should define the maximum time for all possible storage conditions that can occur in routine practice - room temperature (20-25 °C), refrigerator (2-8 °C) and freezer (- 20 °C and below) and for all sample types used (serum, plasma). The storage duration is defined in the manufacturer’s instructions for use, but the MBLs can choose other literature data if the source of data is documented through proper documentation. This recommendation is useful in establishing acceptable delays and storage conditions when immediate processing of samples is not feasible.

## Analytical recommendations

8. The MBL should verify the thyroid function test measurement method before introducing it into routine practice.

The analytical verification protocol should include at least an assessment of precision, method comparison (if available), and measurement uncertainty. The most widely used guidelines are published by the Clinical and Laboratory Standards Institute (CLSI), specifically CLSI EP15 for precision, and CLSI EP09 for method comparison using patient samples (*56*, *57*). Measurement uncertainty should be estimated from data obtained during the verification procedure or long-term quality control (QC) data, as recommended by the joint Working group for measurement uncertainty of the CSMBLM and the CCMB (*58*). In conclusion, rapid technological advancements can impact procedures, requiring laboratories to regularly verify TFTs and update their protocols following the latest available guidelines.

9. The limit of quantitation should be used as the lowest reportable limit for TSH and Tg.

10. The MBL should verify the manufactur-er-claimed limit of quantitation before im-plementing it in routine work.

New generations of TSH and Tg assays significantly lowered the limit of quantification (LOQ), improving detection of patients with subclinical hyperthyroidism or follow-up of patients with differentiated thyroid cancer (DTC) (*59*, *60*). Almost 60% of the participants of the survey declared to use the limit of detection (LOD) as the lowest value of reported TSH, while about 40% use the LOQ (*27*). A few participants performed verification protocol for LOQ claimed by the manufacturer (*27*). Since medical decision points are determined around LOQ, the laboratory must verify LOQ based on the manufacturer’s claims and, optionally, over time, assess its own (*60*, *61*). The experimental design is described in CLSI EP17 and requires a reference standard with assigned target values (*62*). The alternative method to confirm the manufacturer’s claims is based solely on a precision goal described in CLSI EP15 (*56*).

11. Internal quality control material should cover a clinically significant range.

12. Internal quality control material should ensure monitoring of TSH and Tg in low con-centrations.

Internal QC should follow the MBL’s established quality management strategies (*63*). The internal QC should cover a clinically significant span of values, including concentrations near the clinical decision limit (cut-off values). As medical decisions rely on the low values of TSH and Tg, the control material must encompass low concentrations to guarantee the accuracy and reliability of test results in that range. If the internal QC provided by the manufacturer does not meet these criteria, the laboratory should consider third-party control materials or retesting of retained patient samples. As a result, MBLs must manage data from internal QC on an ongoing basis, regularly assess performance, and compare it against the acceptance criteria set by the laboratory.

13. The MBL should participate in an external quality assessment scheme for all performed thyroid function tests, and the assay perfor-mance must meet the minimum criteria.

Croatian MBLs must participate in the external quality assessment (EQA) organized by the Croatian centre for quality assessment in laboratory medicine. The program covers general TFTs, except anti-TPO, anti-Tg, and anti-TSHR. Some MBLs performing antibody testing are not participating in EQA schemes (*27*). If these tests are used in routine practice, the MBL must ensure the availability of alternative EQA organized by other international providers at least once a year. Testing should be conducted using the same procedures for patient samples. Any deviation based on the EQA provider’s evaluation must be analyzed and recorded *per* laboratory policies.

14. Thyroglobulin should always be measured with anti-Tg, and both results should be available on the laboratory report.

15. To categorize patients with differentiated thyroid cancer as anti-Tg-positive, it is pref-erable to use the limit of detection, the limit of quantification, or a method-specific cut-off rather than the reference interval for a healthy population.

16. The laboratory report should include a comment on possible interference when re-porting Tg values in anti-Tg-positive patients.

Some patients present with positive anti-Tg antibodies at their initial diagnosis, which may remain in circulation for an extended period and potentially interfere with Tg measurement (*64*). The lack of analytical comparability between different assay methods requires consistent follow-up using the same analytical platform to ensure reliable longitudinal assessment. The presence of anti-Tg in the sample can significantly impact the interpretation of Tg test results in patients with DTC due to mostly negative interference. There are several possibilities to determine or circumvent the anti-Tg interference: (i) measuring anti-Tg by immunoassays, (ii) determining the Tg concentration through a recovery test, or (iii) measuring Tg with liquid chromatography-tandem mass spectrometry (LC-MS/MS) directly (*65*). Whether the sample is classified as anti-Tg-positive or anti-Tg-negative depends on the specific immunoassay, assay sensitivity, extent of interference by endogenously occurring Tg, and anti-Tg heterogeneity (*66*, *67*). The presence of anti-Tg itself does not necessarily indicate interference and the level of interference is poorly correlated with the concentrations of anti-Tg. To assess interference, the Tg recovery test was created as an interaction between endogenous and added Tg with anti-thyroglobulin antibodies. However, the recovery test did not demonstrate clinical benefits due to technical constraints and the inability to adjust Tg concentrations in the presence of anti-thyroglobulin antibodies (*68*). As an alternative approach, LC-MS/MS has been identified as the preferred method for assessing Tg values in the presence of interference from anti-Tg. Although this method should be the choice for anti-Tg-positive patients, it lacks sensitivity and robustness and is not widely accessible in Croatian MBLs (*69*-*71*).

Automated immunoassays are the most commonly used methods for anti-Tg and Tg measurement to follow up on DTC patients. It is preferable to use LOD, LOQ, or a method-specific cut-off to categorize patients with DTC as anti-Tg-positive. Lowering the threshold at the LOD or LOQ level increases the number of anti-Tg-positive test results. The laboratory can implement a method-specific cut-off as an alternative option to reduce the number of false positive test results.

**Figure 1:**
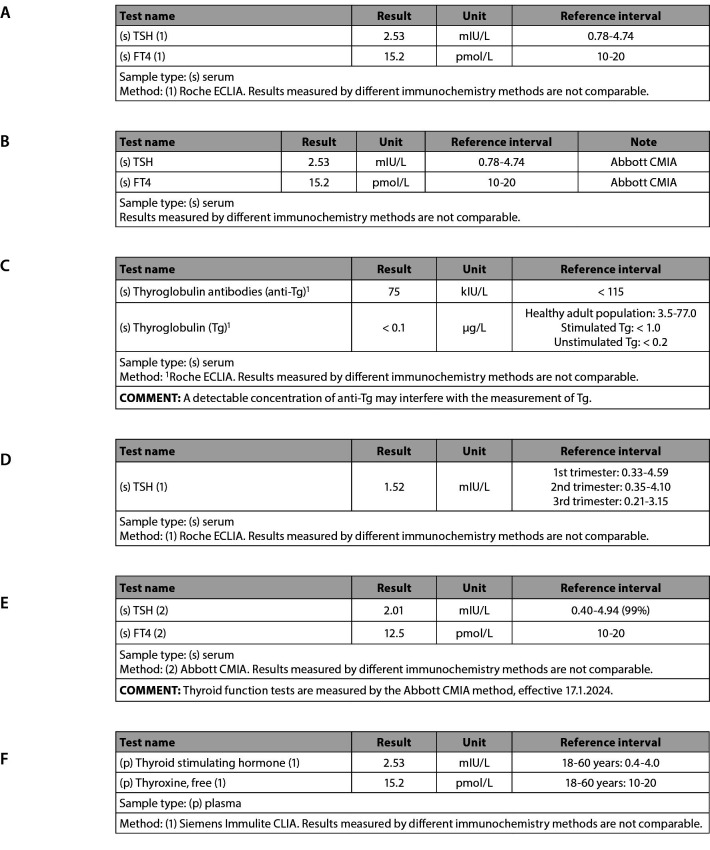
Examples of laboratory reports. Reference intervals in this figure are for example purposes only. The MBL can choose different reporting options, depending on the possibilities of the laboratory information system and organization. A) Reporting on assay method. The assay method is coded with the number 1 and a legend at the bottom. The report warns that different assays can yield different results. B) Reporting on assay method. The assay method is next to each result in the Note field. The report warns that different assays can yield different results. C) Reporting on the potential interference of thyroglobulin antibodies (anti-Tg) when measuring thyroglobulin. Although anti-Tg is within the reference range for a healthy population, the measured concentration is above the method’s limit of quantification, and inference is possible. D) Reporting on trimester- and method-specific reference intervals in pregnancy. The example shows 95% method-specific reference intervals by trimester. E) Reporting on the assay method change. The laboratory changed the assay method on 17.1.2024 and reports on the change for the next 6 months. The new method for TSH has defined a reference interval with 99% width as opposed to the recommended 95% width. Therefore, a comment is included on the report next to the reference interval for TSH. F) Reporting on reference intervals for the closest age group available. The patient’s age is 70, and no age-appropriate reference interval is defined in the laboratory. The closest reference interval is for the 18-60 age group, and therefore, there is a note on the report next to the reference interval for TSH and FT4.

The concentration of Tg should always be measured with anti-Tg, and both results should be visible to the physician on the laboratory report. Failure to report Tg values in anti-Tg-positive patients would lead to a significant number of unreportable results, as between 8 and 55% of DTC patients are anti-Tg-positive (depending on the measurement method and cut-off value used) (*66*). If the patient is anti-Tg-positive, the report should include a comment on possible interference (Figure 1).

17. The MBL should establish protocols to confirm the presence of interfering substances.

18. In case of confirmed interference, the MBL should inform clinicians, and the test result should not be reported. Instead, it should include a comment on their presence.

Interferences affect the results obtained with all immunoassays; the most common are hemolysis, icterus, and lipemia (HIL). Hemolysis, icterus, and lipemia indices are typically measured on an automated clinical chemistry platform by spectrophotometry (*72*, *73*). Acceptance criteria for these measurements are usually derived from manufacturers’ specifications. The laboratory must implement a protocol for measuring HIL interference and define acceptance criteria for TFT.

Interference can also originate from human anti-animal antibodies, human anti-mouse antibodies, and heterophilic antibodies, as well as macro-TSH, biotin, anti-streptavidin antibodies, anti-ruthenium antibodies, and thyroid hormone autoantibodies. Although prevalence of these interferences is low, they can lead to misdiagnosis or inappropriate management (*74*). Only a small proportion of Croatian MBLs have implemented protocols for samples under suspicion of analytical interference, thus leaving them under-recognized (*27*). If there are discordances in clinical findings and test results or any suspicion of their presence, the laboratory should establish clear protocols for sample handling. Confirmed interferences should have interpretative comments on the patient report (e.g. *The TSH test result cannot be reported due to suspicion of interference*). It is important to note that a single test is usually insufficient to identify interferences. Combining procedures such as preanalytical factors checkup, additional laboratory tests, method comparison, serial dilutions, polyethylene glycol precipitation, sample treatment with a heterophilic blocking tube, measuring rheumatoid factor and size exclusion chromatography can be helpful in these cases. However, negative tests do not necessarily rule out interferences. Laboratory personnel can investigate patients’ recent immunization procedures, transfusions, autoimmune diseases, monoclonal therapy, contact with pets, or occupational exposure as potential sources of interference (*74*, *75*).

19. Laboratory specialists should be aware of certain medications that can affect the results of thyroid function tests and binding protein abnormalities. They should use this knowledge to consult physicians to interpret test results correctly.

Certain medications can affect TFTs. As for the interferences described above, medication must be included as a potential source of discrepancies. Medications can interfere with the production, release, transportation, and metabolism of thyroid hormones and the absorption of thyroid hormone therapy. Some medications can have multiple effects on thyroid hormone concentrations, and these effects can change in different directions during treatment. A list of the most commonly used drugs that can interfere with TFT can be found in Table 1.

**Table 1:** Effects of various medications on thyroid function tests

**Mechanism (References)**	**Medication**	**Effect**
Inhibit TSH secretion (*97*, *98*)	Bexarotene Dobutamine Dopamine and dopamine agonists Glucocorticoids Immune checkpoint inhibitors L-dopa Somatostatin	¯ TSH ¯ T3 ¯ T4
Stimulation of TSH secretion (*99*)	Amisulpiride	TSH
Inhibition of thyroid hormone production or release (*98*)	Iodine Lithium	TSH ¯ T3 ¯ T4
Inhibit conversion T4 to T3 (*98*)	Amiodarone Glucocorticoids Propranolol Radiocontrast agents	« TSH ¯ T3 ¯ « T4 ¯ « FT4
Inhibit gastrointestinal absorption of T4 (*97*, *98*)	Aluminum hydroxide Antacids Calcium salts Cholestyramine Colestipol Ferrous sulphate Kayexalate Proton pump inhibitors Soybean preparations	TSH ¯ T4 ¯ FT4
Inhibit binding of T4 or T3 to serum proteins (*98*)	Carbamazepine Furosemide Heparin (*in vitro*) Nonsteroidal anti-inflammatory drugs Phenytoin Salicylate	« TSH ¯ T3 ¯ T4 « FT4
Increase in T4 and T3 metabolism or clearance (*98*, *100*)	Carbamazepine Phenobarbital Phenytoin Rifampicin Ritonavir	« TSH ¯ T4 ¯ FT4
TBG excess (*97*, *98*)	Oestrogens Clofibrate 5-fluorouracil Opiates Perphenazine Tamoxifen	« TSH T3 T4 « FT4
TBG suppression (*97*, *98*)	Anabolic steroids Androgens Glucocorticoids	« TSH ¯ T3 ¯ T4 « FT4
Hypothyroidism preceded by destructive thyroiditis (*101*)	Immune checkpoint inhibitors Interferon alfa Interleukin-2 Tyrosine kinase inhibitors	TSH ¯ T3 ¯ T4 ¯« FT4
Thyrotoxicosis (*101*)	Immune checkpoint inhibitors Interferon alfa Interleukin-2 Iodine and iodide-containing drugs Tyrosine kinase inhibitors	¯ TSH T4
TSH - thyroid stimulating hormone. T3 - total triiodothyronine. T4 - total thyroxine. FT4 - free thyroxine. TBG - thyroxine binding globulin. - increase in concentration. ¯ - decrease in concentration. « - no change in concentration.		

## Postanalytical recommendations

20. The MBL should implement method- and age-specific reference intervals appropriate for the Croatian population.

It is the MBL’s responsibility to report reference intervals (RIs) appropriate to the tested population, bearing in mind the analytical method used to measure the concentration. Most Croatian MBL use literature data for RIs, such as manufacturer’s kit inserts or other published data (*27*). Regardless of the literature source, the laboratory must critically review reference intervals - similarity of population, number of subjects, age stratification, statistics and analytical method - before clinical implementation.

The concentration of TFTs changes with age, and these alterations are more pronounced at both ends of the life span. Survey results showed that half of participating Croatian MBLs do not use RIs for children (*27*). Multiple researchers have established RIs for thyroid hormones in pediatric population (*76*-*79*). However, results are influenced by Tanner stage, ethnicity, anthropometric characteristics, iodine intake and method differences, which result in considerable between-study heterogeneity in the established pediatric RIs. Despite between-study heterogeneity in results, MBLs should strive to implement age-specific RIs for children to improve clinical outcomes. Recent evidence shows that different RIs also need to be adapted in the elderly as regulation and baseline TSH concentration undergo significant changes with age. In individuals over 60 years, especially in women, TSH concentration tends to increase (*80*). The European Thyroid Association recommends age-specific RIs for TSH to establish a diagnosis of subclinical hypothyroidism in older people (*20*). Multiple studies observed an age-related increase in TSH and proposed new RIs tailored to specific age groups. This approach acknowledges the physiological changes in TSH concentrations that occur with ageing and avoids unnecessary treatment in the elderly (*21*, *80*-*83*).

Therefore, we recommend that the laboratory uses age-appropriate RIs for children, adults and the elderly population. If RIs for children or the elderly are not available, (i) the result report should contain a comment that RIs are not appropriate for the patient’s age, or (ii) the result report should provide RIs of the closest age group (*84*) (Figure 1).

21. Trimester-related, population- and meth-od-specific reference intervals should be available for pregnant women and their physi-cians.

The thyroid adapts during pregnancy to meet increased metabolic needs. Maternal thyroid is stimulated in the first trimester by human chorionic gonadotrophin, resulting in lower concentrations of TSH during early pregnancy compared to non-pregnant women. Pregnancy is also characterized by an increased iodine renal clearance, increased serum TBG concentration, and inner-ring deiodination of T3 and T4 by the placenta (*22*). These metabolic changes influence the T3 and T4 concentrations, which appear to increase during the first trimester and decrease relatively during the second and third trimesters. Modern laboratories utilize automated indirect analog immunoassays to measure FT4 concentrations, which are affected by the increase in TBG and decrease in albumin concentrations during pregnancy. During the third trimester, there is method dependent reduction in the measured FT4 concentration (*85*, *86*). As a result, TFT results of healthy pregnant women differ from those of non-pregnant women. This calls for trimester-specific RIs for all TFTs, especially for the most commonly used TSH and FT4, keeping in mind population and method differences. Used RIs should be established in pregnant women with no known thyroid disease, with optimal iodine intake, and negative anti-TPO (*23*). Trimester- and population-specific RIs are recommended as the primary reporting method by various international guidelines. (*22*, *23*, *25*, *26*). Universal cut-offs (such as 2.5 or 4 mIU/L) should only be used if adequate RIs are not established for the population of interest. It is important to distinguish between RIs used to diagnose thyroid disease in pregnancy and the specific treatment targets for women using thyroid-related medication. Treatment targets should not be used as pregnancy RIs when testing is performed to diagnose thyroid dysfunction (*25*). If the laboratory chooses to use universal cut-offs instead of method- and population-specific RIs, this should be noted on the report and properly communicated with physicians and patients. The best course of action would be to print RIs by the trimester on the laboratory report (Figure 1), but this is often technically not possible, nor does the laboratory personnel know if the patient is pregnant. At a minimum, the MBL should define method-specific RIs for pregnant women by trimester (at least for TSH and FT4), and RIs should be available for the patients and their physicians on request (*e.g.* through comments on the report or web page).

22. The MBL should use 95% reference inter-vals (defined by 2.5th and 97.5th percentiles). If any other reference interval is used, this should be stated on the report.

By convention, a reference range usually comprises a 95% interval bounded by the 2.5^th^ and 97.5^th^ percentiles. Thus, 2.5% of “normal” individuals will fall above the reference range, and 2.5% will fall below the range (*24*, *87*). However, some laboratories use a wider 99% RI for TSH, which especially affects the upper limit of RI, shifting it to higher values (*27*). As RIs are method-dependent, introducing another variation in the form of a different RI width is not advisable. Medical-biochemistry laboratories should report 95% RIs, and if (and only if) 95% are not available, they should use other RI widths (*e.g.* 90% or 99%) accompanied by a comment on the report (Figure 1). As anti-Tg, anti-TPO, and anti-TSHR are usually defined only with the upper reference limit, no additional comment is necessary. However, antibodies can be reported with cut-off values for diseases (*e.g.* Graves), rather than RIs derived from a healthy population. If cut-off values are used, this should be noted on the report.

23. The report should include recommended Tg cut-off values for follow-up patients with differentiated thyroid carcinoma.

Thyroglobulin is mainly used as a tumor marker in patients with DTC. The RIs provided by manufacturers are intended for the general healthy population and are not suitable for DTC patients. Experts and national guidelines suggest specific Tg cut-offs when using highly sensitive Tg assays. Proposed cut-offs categorize the risk of recurrence in follow-up patients (*61*, *88*, *89*). In addition to RIs for the general population, the report should include Tg cut-off values for DTC patients who have undergone total thyroidectomy, with or without radioiodine therapy, as suggested by expert committees. The MBL should discuss the Tg cut-off with clinicians, which should be reported together with RIs for the healthy population. Cut-off values can be defined with one or more of the following: (i) unstimulated (basal) Tg for low risk of recurrence; (ii) stimulated Tg for low risk of recurrence; (iii) DTC risk stratification - low, medium or high risk based on unstimulated (basal) Tg; (iv) DTC risk stratification - low, medium or high risk based on stimulated Tg. Regardless of the literature source or type of cut-off values used, the MBL should document it appropriately.

24. The laboratory report should include the manufacturer and short method name as pre-sented in Table 2.

Croatian MBLs use at least eight different analytical methods to measure the concentration of TSH, as seen in the survey (*27*). As harmonization and standardization of TFTs are still not available, there are differences in measured concentrations when using different analytical methods. Therefore, the MBL should report the manufacturer’s name coupled with a short method name (*e.g.* Abbott CMIA). The analyzer name should be reported if the same manufacturer uses more than one type of method (*e.g.* Siemens Immulite CLIA and Siemens Atellica CLIA). Table 2 summarizes the most frequent names used in Croatian MBLs (*27*). We advise against using the full name of the method (*e.g.* full name of CMIA – chemiluminescent microparticle immunoassay) as interchangeable use of long and short names could add to confusion for the non-laboratory personnel reading the report. For the same reason, we also advise against using specific types of analyzers (*e.g.* Roche cobas e411 ECLIA). Examples are shown in Figure 1.

**Table 2:** The most common manufacturer and method names

**Manufacturer and short method name**	**Long (full) method name**
Abbott CMIA	chemiluminescent microparticle immunoassay
Beckman Coulter CLIA	chemiluminescent immunoassay
Roche ECLIA	electrochemiluminescent immunoassay
Siemens Atellica CLIA	chemiluminescent immunoassay
Siemens Immulite CLIA	chemiluminescent immunoassay
Snibe CLIA	chemiluminescent immunoassay
Tosoh FEIA	fluorescence enzyme immunoassay
CMIA - chemiluminescent microparticle immunoassay. CLIA - chemiluminescent immunoassay. ECLIA - electrochemiluminescent immunoassay. FEIA - fluorescence enzyme immunoassay. Abbott Diagnostics, Santa Clara, USA. Beckman Coulter, Brea, USA. Roche Diagnostics GmbH, Mannheim, Germany. Tosoh Corporation, Tosoh, Japan. Siemens Healthcare GmbH, Erlangen, Germany. Snibe - Shenzhen New Industries Biomedical Engineering, Shenzhen, China.	

25. The laboratory report should include comments on differences between assay re-sults. If the laboratory changes the assay method, the report should also include a comment describing the change for at least six months.

As immunoassays can measure concentrations differently, the CCMB requires that each report with immunoassay test results should include a comment on assay incomparability (*90*). We agree with this approach, and reports containing TFT results should include a comment so that physicians are continuously reminded of this fact. With that in mind, each change in assay method (*e.g.* from Roche ECLIA to Abbott CMIA) should be additionally communicated with the users through a comment on the report for at least six months after the switch (Figure 1).

26. Analyte should be stated with full name and/or internationally accepted abbreviations, and results should be reported in SI units as presented in Table 3.

To avoid possible misunderstandings in the interpretation of the results, it is desirable to state (i) only the full name of the analyte, (ii) only the abbreviation or (iii) both, as listed in Table 3 (*91*). Further, a very common issue is the reporting units for laboratory tests. There are two different unit systems: conventional units and Système International d’Unités (SI) units. In 1966, the International Federation of Clinical Chemistry and Laboratory Medicine recommended the use of SI units for the clinical laboratory: whenever possible, use mol and liter units to express concentration. This approach was designed to provide a clearer quantitative relationship among molecular species and some standardization of databases (*92*).

**Table 3:** Full name of thyroid function tests with abbreviations and units

**Full name**	**Abbreviation**	**Unit**
Thyroid stimulating hormone	TSH	mIU/L
Triiodothyronine, total	T3	nmol/L
Thyroxine, total	T4	nmol/L
Triiodothyronine, free	FT3	pmol/L
Thyroxine, free	FT4	pmol/L
Thyroglobulin antibodies	anti-Tg	kIU/L
Thyroid peroxidase antibodies	anti-TPO	kIU/L
Thyroid stimulating hormone receptor antibodies	anti-TSHR	kIU/L
Thyroglobulin	Tg	µg/L

27. Each MBL should define its own critical values according to expected disease preva-lence.

The concept of critical value reporting was first postulated by Lundberg in the 1970s. Since then, critical values have been adopted in laboratories all over the world as an essential tool that must be communicated to the responsible/treating physician (*93*, *94*). In recent years, many surveys have examined the best way to define cut-off values, assess responsibility for communicating critical values, and identify indicators to monitor process improvement. The CCMB and Lenicek Krleza *et al.* give an overview of critical values but also state that each MBL should define its own critical values in the context of the clinical importance and harmonize them with the physician’s need in the correct assessment of the patient’s condition and timely medical care (*95*, *96*). It is important to note that parameters and values chosen as critical limits depend essentially on the disease prevalence expected in the clinic or practice. The source of critical values (*e.g.*, CCMB recommendation) should be noted in proper documentation. The MBL can choose not to define critical values for TFTs, but this decision should be explained and documented.

## Appendix 1. Summary of recommendations

**Table:** 

**PREANALYTICAL RECOMMENDATIONS**
1. The manufacturer’s instructions for the sample matrix should be followed.
2. Blood samples for thyroid function tests should be taken in fasting state.
3. Patients should refrain from strenuous physical activity 24 hours before blood sampling for thyroid function tests.
4. Blood samples should be collected between 7 and 11 AM due to the circadian rhythm of TSH and FT3.
5. Any thyroid replacement or suppression therapy should be administered after blood collection.
6. Patients should avoid any therapeutic and diagnostic procedures affecting thyroid function tests 1 to 3 months before blood collection.
7. The MBL should define analyte stability time for thyroid function tests.
**ANALYTICAL RECOMMENDATIONS**
8. The MBL should verify the thyroid function test measurement method before introducing it into routine practice
9. The limit of quantitation should be used as the lowest reportable limit for TSH and Tg.
10. The MBL should verify the manufacturer-claimed limit of quantitation before implementing it in routine work.
11. Internal quality control material should cover a clinically significant range.
12. Internal quality control material should ensure monitoring of TSH and Tg in low concentrations.
13. The MBL should participate in an external quality assessment scheme for all performed thyroid function tests, and the assay performance must meet the minimum criteria.
14. Thyroglobulin should always be measured with anti-Tg, and both results should be available on the laboratory report.
15. To categorize patients with differentiated thyroid cancer as anti-Tg-positive, it is preferable to use the limit of detection, the limit of quantification, or a method-specific cut-off rather than the reference interval for a healthy population.
16. The laboratory report should include a comment on possible interference when reporting Tg values in anti-Tg-positive patients.
17. The MBL should establish protocols to confirm the presence of interfering substances.
18. In case of confirmed interference, the MBL should inform clinicians, and the test result should not be reported. Instead, it should include a comment about their presence.
19. Laboratory specialists should be aware of certain medications that can affect the results of thyroid function tests and binding protein abnormalities. They should use this knowledge to consult physicians to interpret test results correctly.
**POSTANALYTICAL RECOMMENDATIONS**
20. The MBL should implement method- and age-specific reference intervals appropriate for the Croatian population.
21. Trimester-related, population- and method-specific reference intervals should be available for pregnant women and their physicians.
22. The MBL should use 95% reference intervals (defined by 2.5th and 97.5th percentiles). If any other reference interval is used, this should be stated on the report.
23. The report should include recommended Tg cut-off values for follow-up patients with differentiated thyroid carcinoma.
24. The laboratory report should include the manufacturer and short method name as presented in Table 2.
25. The laboratory report should include comments on differences between assay results. If the laboratory changes the assay method, the report should also include a comment describing the change for at least six months.
26. Analyte should be stated with full name and/or internationally accepted abbreviations, and results should be reported in SI units as presented in Table 3.
27. Each MBL should define its own critical values according to expected disease prevalence.
MBL - medical-biochemistry laboratory.

## Data Availability

No data was generated during this study, so data sharing statement is not applicable to this article.
